# Equine Anti-SARS-CoV-2 Serum (ECIG) Binds to Mutated RBDs and N Proteins of Variants of Concern and Inhibits the Binding of RBDs to ACE-2 Receptor

**DOI:** 10.3389/fimmu.2022.871874

**Published:** 2022-07-11

**Authors:** Sonia Aparecida Andrade, João Victor Batalha-Carvalho, Rui Curi, Fan Hui Wen, Dimas Tadeu Covas, Ana Marisa Chudzinski-Tavassi, Ana Maria Moro

**Affiliations:** ^1^ Biopharmaceuticals Laboratory, Instituto Butantan, São Paulo, Brazil; ^2^ University of São Paulo, Sao Paulo, Brazil; ^3^ Cruzeiro do Sul University, São Paulo, Brazil; ^4^ Immunobiological Production Section, Bioindustrial Center, Butantan Institute, São Paulo, Brazil; ^5^ Directory, Directory Instituto Butantan, São Paulo, Brazil; ^6^ Center of Excellence in New Target Discovery (CENTD), Instituto Butantan, São Paulo, Brazil; ^7^ Innovation and Development Laboratory, Instituto Butantan, São Paulo, Brazil; ^8^ Center for Research and Development in Immunobiologicals (CeRDI), Instituto Butantan, São Paulo, Brazil

**Keywords:** COVID-19, equine serum, neutralizing antibodies, nucleocapsid, RBD, VOCs, SPR

## Abstract

The COVID-19 pandemic caused by the severe acute syndrome virus 2 (SARS-CoV-2) has been around since November 2019. As of early June 2022, more than 527 million cases were diagnosed, with more than 6.0 million deaths due to this disease. Coronaviruses accumulate mutations and generate greater diversity through recombination when variants with different mutations infect the same host. Consequently, this virus is predisposed to constant and diverse mutations. The SARS-CoV-2 variants of concern/interest (VOCs/VOIs) such as Alpha (B.1.1.7), Beta (B.1.351), Gamma (B.1.1.28/P.1), Delta (B.1.617.2), and Omicron (B.1.1.529) have quickly spread across the world. These VOCs and VOIs have accumulated mutations within the spike protein receptor-binding domain (RBD) which interacts with the angiotensin-2 converting enzyme (ACE-2) receptor, increasing cell entry and infection. The RBD region is the main target for neutralizing antibodies; however, other notable mutations have been reported to enhance COVID-19 infectivity and lethality. Considering the urgent need for alternative therapies against this virus, an anti-SARS-CoV-2 equine immunoglobulin F(ab’)_2_, called ECIG, was developed by the Butantan Institute using the whole gamma-irradiated SARS-CoV-2 virus. Surface plasmon resonance experiments revealed that ECIG binds to wild-type and mutated RBD, S1+S2 domains, and nucleocapsid proteins of known VOCs, including Alpha, Gamma, Beta, Delta, Delta Plus, and Omicron. Additionally, it was observed that ECIG attenuates the binding of RBD (wild-type, Beta, and Omicron) to human ACE-2, suggesting that it could prevent viral entry into the host cell. Furthermore, the ability to concomitantly bind to the wild-type and mutated nucleocapsid protein likely enhances its neutralizing activity of SARS-CoV-2. We postulate that ECIG benefits COVID-19 patients by reducing the infectivity of the original virus and existing variants and may be effective against future ones. Impacting the course of the disease, mainly in the more vulnerable, reduces infection time and limits the appearance of new variants by new recombination.

## Introduction

The severe acute syndrome virus 2 (SARS-CoV-2) is an RNA virus with higher transmission rates when compared to earlier coronavirus outbreaks (e.g., SARS and MERS) (1). As an evolutionary mechanism, coronaviruses accumulate mutations through recombination when different variants infect the same host or when the virus persists for a long time in the host, resulting in greater viral diversity (2). Consequently, this virus is predisposed to constant and diverse mutations. The SARS-CoV-2 genome comprises approximately 30,000 nucleotides, encoding four structural proteins: spike (S), envelope (E), membrane (M), and nucleocapsid (N) (3). The S protein is a trimeric glycoprotein containing 1273-amino acid. The monomers are composed of two domains: S1 (residues 1-686) and S2 (residues 687-1273). The S1 domain is exposed and contains the receptor-binding domain (RBD) (residues 306-534), while the S2 domain is partially buried (3, 4). New variants of SARS-CoV-2 have been identified with alterations in the S protein. For example, the Alpha (B.1.1.7), Beta (B.1.351), Gamma (B.1.1.28/P.1), Delta (B.1.617.2), Kappa (B.1.617.1), and Omicron (B.1.1.529), which was classified as a variant of concern (VOC) by the World Health Organization (WHO) on November 2021, display S protein mutations and have quickly spread across the world (1, 4–6). A previous study demonstrated that the N501Y mutation, present in all VOCs except for Delta, exhibits increased ACE-2 binding affinity, conferring higher resistance to neutralization (7). The E484K mutation observed in Gamma, Beta, and Omicron (as E484A) can evade neutralization by most monoclonal antibodies and reduce mRNA vaccine responses (6–9). Another significant VOC mutation occurs at amino acid 417. A K417T was reported in the Gamma variant, but in Beta and Omicron, the lysine is substituted by asparagine (K417N) (4, 8). It should be pointed out that the K417N/T mutations rarely occur in the absence of other mutations, possibly because K417 mutations appear to reduce ACE-2 binding. Additionally, the L452R mutation is present in the Delta, a VOC, and in Kappa (B.1.617.1) and Epsilon (B.1.427/9), which are variants of interest (VOIs). This mutation reduces the susceptibility of the S protein to several Region Binding Motif (RBM) class 2 monoclonal antibodies (mAb). In one study, the samples with the L452R mutation displayed 3 to 10-fold reduced susceptibility to about one-third of plasma samples from convalescent and vaccinated individuals (4, 10). The mutations described above are of concern because they are located in the region interacting with the angiotensin-2 converting enzyme (ACE-2), the main route of human SARS-CoV-2 infection. Thus, it should be no surprise that this region has become the main target for neutralizing antibodies.

Besides RBD mutations, others have also been found in the S1 domain and S2 domain, as the D614G, present in the S1 domain, impacts identified sample frequency (IF) and increases fitness, infectivity, and fatality (11). In addition to the S protein, SARS-CoV-2 infected patients also present an early antibody response against the N protein present within the viral particle, not on the surface (12, 13). The SARS-CoV-2 N protein contains two distinct RNA-binding domains: the N-terminal domain (NTD) and the C-terminal domain (CTD). These two domains are linked by a poorly structured linkage region (LKR) containing a serine/arginine-rich (SR-rich) domain (SRD) (14, 15). It has been proposed that NTD and CTD bind to the viral RNA genome through electrostatic interaction and are essential to the virus life cycle. Mutations in the N protein have been detected, and it is plausible that they functionally contribute to the virulence of the virus. For example, the co-occurring R203K/G204R mutations in the N protein rapidly increase in frequency and are closely associated with the virus’ infectivity (16). Indeed, these mutations are carried by Alpha (B.1.1.7) (17, 18), Gamma (B.1.1.28/P.1) (19, 20), and Omicron variants (6).

Given the importance of other structural proteins for the SARS-CoV-2 life cycle, antibodies that inhibit or interfere with these proteins may effectively reduce infections’ severity and subsequent consequences, like hospitalization and death. Considering the urgent need for alternative COVID-19 therapies, developing antibodies that recognize and bind to N-protein mutations concomitantly to RBD could be a viable and effective strategy for treating COVID-19-infected patients. In the present study, the Butantan Institute created, through immunization of horses using inactivated viruses (SARS-CoV-2/SP02/2020HIAE - GenBank MT126808.1. B.1.1.28), an equine Covid immunoglobulin F(ab’)2, herein referred to as ECIG). This method employed multiple cycles of subcutaneous inoculation without harm, as the virus was inactivated by gamma-irradiation, yielding large amounts of plasma and homogeneous final preparations of highly purified antibodies (21). A Phase I/II Randomized Clinical Trial approved by the Brazilian Health Regulatory Agency (ANVISA) (https://clinicaltrials.gov/ct2/show/NCT04834089) is ongoing to evaluate ECIG’s safety pharmacokinetics and efficacy in patients with an increased risk for severe disease and at an early stage of COVID-19 infection.

Surface Plasmon Resonance was utilized to evaluate ECIG binding to wild-type and mutated RBD, Spike, and N proteins. The results demonstrated that ECIG generated by injecting inactivated, yet structurally preserved whole virus, could successfully bind to mutated viral RBD, Spike, and N proteins. The affinity and binding of ECIG to the virus’ proteins indicate that this therapy could minimize and/or inhibit critical SARS-CoV-2 interactions essential for viral entry *via* ACE-2, the viral life cycle, and its replication. Thus, ECIG appears to be a promising therapeutic alternative for treating individuals infected with COVID-19 caused by wild-type SARS-CoV-2 and presently known variants. Besides, ECIG could also be effective against future variants.

## Material and Methods

### Material

SARS-CoV-2 (2019-nCoV) Spike RBD Recombinant proteins (RBD wild type), RBD (E484K), RBD (N501Y), RBD Beta, Gamma, Delta, Omicron, S1+S2 Gamma, Delta, Omicron and Nucleocapsid recombinant proteins (N/wild type and N mut/del), and ACE-2 protein were purchased from Sino Biological (Chesterbrook, PA, USA). RBD, N wild type (WU), and S1+S2 Gamma proteins were expressed in insect cells, N mut/del was expressed in *E.coli*, and all the other commercial proteins, including ACE-2, were expressed in HEK293 cells). HBS-EP buffer (0.01 M HEPES pH 7.4, 0.15 M NaCl, 3 mM EDTA, 0.005% v/v Surfactant P20) was used in SPR experiments. ECIG was purified by the Immunobiological Production Section, Bioindustrial Center, Butantan Institute from the plasma of horses immunized with inactivated SARS-CoV-2 virus, collected up to 49 days after the first antigen injection. The final product is composed of F(Ab’_2_) immunoglobulin fragments (21).

### Cloning and Expression of RBD Gamma and Delta in CHO Cells

Two SARS-CoV-2 RBD sequences were designed as homodimers of RBD Gamma (RBDγ) with K417T/E484K/N501Y mutations and RBD Delta plus (RBDδp) with K417N/L452R/T478K mutations. The constructs were synthesized by GeneArt (ThermoFisher Scientific – Invitrogen) and contained an N-terminal SARS-CoV-2 S protein sequence signal, a C-terminal AviTag sequence, and a 6×HisTag. Plasmids were transformed into *Escherichia coli* One Shot TOP10 (Invitrogen, C404003, City, State, Country) by heat shock. A randomly selected colony-forming unit (CFU) was propagated. Following cell lysis, the plasmids were purified with the PureLinkTM HiPure Plasmid Maxiprep Kit (Invitrogen, K210007) following the manufacturer’s instructions. The plasmid DNA was then transiently transfected into ExpiCHO cells using the ExpiCHOTM Expression System (Gibco, A29133, City, State, Country) following the manufacturer’s protocol. Standard methodologies were utilized to purify the recombinant antigens with Ni Sepharose 6 Fast Flow resin (Cytiva, 17531801, City, State, Country).

### Binding Analysis

The surface plasmon resonance experiments were performed at room temperature using a GE Biacore T-200 system (GE Healthcare, Chesterbrook, PA, USA). For the binding affinity assays, SARS-CoV-2 RBD, WT; E484K; N501Y; Beta K417N/E484K/N501Y; Gamma (K417T/E484K/N501Y), Delta (L452R/T478K), Delta plus (K417N/L452R/T478K), Omicron (G339D, S371L, S373P, S375F, K417N, N440K, G446S, S477N, T478K, E484A, Q493R, G496S, Q498R, N501Y, Y505H), Gamma Spike S1+S2 (L18F, T20N, P26S, D138Y, R190S, K417T, E484K, N501Y, D614G, H655Y, T1027I, V1176F), Delta Spike S1+S2 (T19R, E156G, 157-158 deletion, L452R, T478K, D614G, F817P, A892P, A899P, A942P, D950N, K986P, V987P), Omicron Spike S1+S2 (A67V, Δ69-70, T95I, G142D/Δ143-145, Δ211/L212I, ins214EPE, G339D, S371L, S373P, S375F, K417N, N440K, G446S, S477N, T478K, E484A, Q493R, G496S, Q498R, N501Y, Y505H, T547K, D614G, H655Y, N679K, P681H, N764K, D796Y, N856K, Q954H, N969K, L981F), and N proteins (WT and the variant containing the D3L/R203K/G204R/S235F mutations) were immobilized on CM5 sensor chips to result in about 1000 resonance units (RU). The reference flow cell was left blank. The running buffer was HBS-EP (0.01 M HEPES pH 7.4, 0.15 M NaCl, 3 mM EDTA, 0.005% v/v Surfactant P20). ECIG and serum samples (1:10 v/v) flowed over the chip surface. After each cycle, the sensor surface was regenerated with 10 mM glycine-HCl pH 2.5. The data were fitted to a 1:1 interaction steady-state binding model using the Biacore T200 Evaluation 3.1 software.

The Committee of Ethics Research in Human Beings approved the serum sample collection (CAAE 3270 7920.0.0000.5467).

### Competition-Binding Study

For competition-binding assays, the ACE-2 protein was diluted in 10 mM sodium acetate buffer, pH 4.5, and then immobilized on the CM5 sensor chip at about 650 RUs. Next, each SARS-CoV-2 RBD (WT, Beta, and Omicron) at gradient concentrations (WT- 100 nM, 200 nM, 300 nM, 400 nM, and for Beta e Omicron: 250 nM, 500 nM, 750 nM, and 1000 nM) flowed over the chip’s surface until achieving the saturation binding between each RBD and ACE-2). Next, at each saturation concentration, competition-binding assays using SARS-CoV-2 RBD (WT, Beta, and Omicron variant) pre-incubated with ECIG [1:10 v/v] for 1 hour at 37°C were also performed. These mixtures were then injected over the surface-immobilized with ACE-2 to evaluate a possible ECIG-mediated inhibition of SARS-CoV-2 RBD binding to ACE-2. After each cycle, the sensor surface was regenerated with 10 mM glycine-HCl pH 2.5.

### SDS PAGE

The purity and molecular mass of commercial and in-house expressed proteins were evaluated by SDS–PAGE at either 7.5% or 12% under nonreducing conditions. Electrophoresis was performed for 90 min at 100 V, and the gel was stained by InstantBlue^®^ Coomassie Protein Stain (Abcam, UK). The molecular weight markers were from Cytiva, Buckinghamshire, UK.

### Statistical Analyses

Experimental data were analyzed using the One-Way ANOVA, followed by the Tukey or Dunnett post-test using the GraphPad Prism program (GraphPad Software, San Diego, CA, USA). P values < 0.05 were considered statistically significant. The values shown in the graphs are presented as means ± SD. One representative result from at least two independent experiments was shown.

## Results

### SARS-CoV-2 Proteins Binding

**Figure 1:**
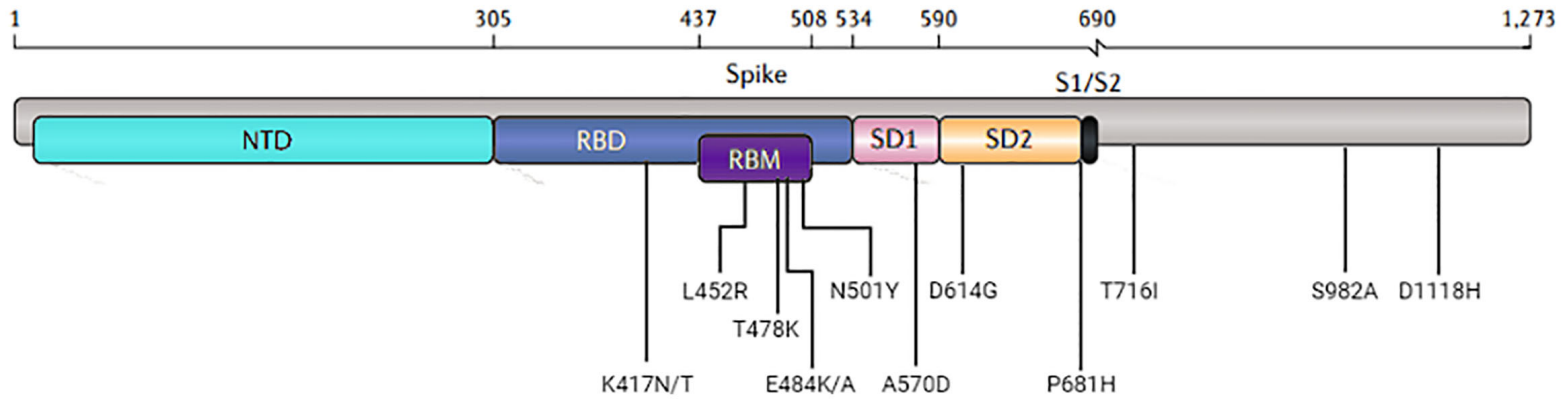
Visual representation of the domain locations and the most common mutations. Abbreviations: NTD, N-terminal domain; RBD, receptor-binding domain; RBM, receptor-binding motif; SD, subdomain; S1/S2, the junction between the exposed S1 attachment domain and the partially buried S2 fusion domain.

**Figure 2:**
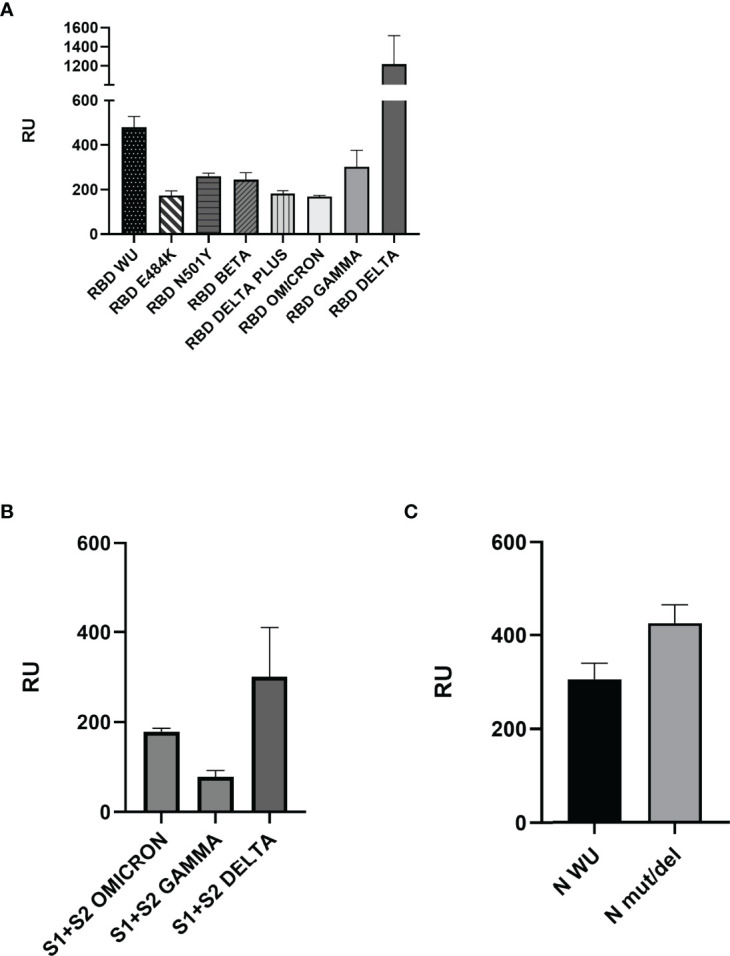
Surface plasmon resonance analyses of ECIG binding to RBD, Spike S1+S2, and N proteins. **(A)** Binding of ECIG to wild-type and mutated RBD. **(B)** Binding of ECIG to Spike S1+S2 Gamma, Delta, and Omicron. **(C)** The binding of ECIG to wild-type N protein and N mut/del. Binding assays were performed with a BIAcore T200 biosensor instrument, with RBD, Spike, and N proteins immobilized on a CM5 sensor chip. Binding responses are represented in resonance units (RU). A running buffer was used as a negative control (0 μM). Results were evaluated by one-way ANOVA and showed statistical significance (p ≤ 0.05).

The notable S protein mutations present in VOCs are presented in **Figure 1**, and the recombinant proteins used in the SPR assays are summarized in summarized in **Table 1**. We checked recombinant proteins’ purity and molecular mass by non-reduced SDS-PAGE (**Supplementary Figure S1**). For the binding assays, ECIG was injected and flowed over the surface of a chip containing recombinant RBD, S1+S2, and N proteins. As shown in **Figure 2A**, the SPR results demonstrated that ECIG binds to wild-type RDB and RBD containing the primary VOC mutations. It was also determined that ECIG binds to S1+S2 ECIG binds to S1+S2 of VOCs (**Figure 2B**) and N protein, WT, and mut/del (**Figure 2C**).

**Table 1:** VOC RBD and N proteins used in the assays.

SARS-CoV-2 Related Proteins	Mutations/deletions	Source	Host	Figure
RBD WU	Original – appeared in China	Sino Biol.	Insect cells	Fig. S1a
RBD E484K	E484K (only mutation)	Sino Biol.	HEK293	Fig. S1a
RBD N501Y	N501Y (only mutation)	Sino Biol.	HEK293	Fig. S1a
RBD Beta	K417N/E484K/N501Y (appeared in South Africa)	Sino Biol.	HEK293	Fig. S1a
RBD Gamma	K417T/E484K/N501Y (appeared in Brazil	Sino Biol.	HEK293	Fig. S1a
RBD Delta	L452R/T478K (appeared in India)	Sino Biol.	HEK293	Fig. S1a
RBD Delta+	K417N/L452R/T478K (appeared in India)	in-house	ExpiCHO	Fig. S1a
RBD Omicron	G339D, S371L, S373P, S375F, K417N, N440K, G446S, S477N, T478K, E484A, Q493R, G496S, Q498R, N501Y, Y505H	Sino Biol.	HEK293	Fig. S1a
S1+S2 Gamma	L18F/20N/P26S/D138Y/R190S/K417T/E484K/N501Y/D614G/H655Y/T1027I/V1176F	Sino Biol.	Insect cells	Fig. S1c
S1+S2 Delta	T19R/E156G/157-158 del./L52R/T478K/D614G/ F817P/A892P/A899P/A942P/D950N/K986P/V987P	Sino Biol.	HEK293	Fig. S1c
S1+S2 Omicron	A67V, Δ69-70, T95I, G142D/Δ143-145, Δ211/L212I, ins214EPE, G339D, S371L, S373P, S375F, K417N, N440K, G446S, S477N, T478K, E484A, Q493R, G496S, Q498R, N501Y, Y505H, T547K, D614G, H655Y, N679K, P681H, N764K, D796Y, N856K, Q954H, N969K, L981F	Sino Biol.	HEK293	Fig. S1c
N WU	N protein of the original virus (Wuhan)	Sino Biol.	Insect cells	Fig. S1b
N mut/del	D3L, R203K, G204R, S235F alterations	Sino Biol.	*E.coli*	Fig. S1b
ACE-2		Sino Biol	HEK293	Fig. S1d

**Figure 3:**
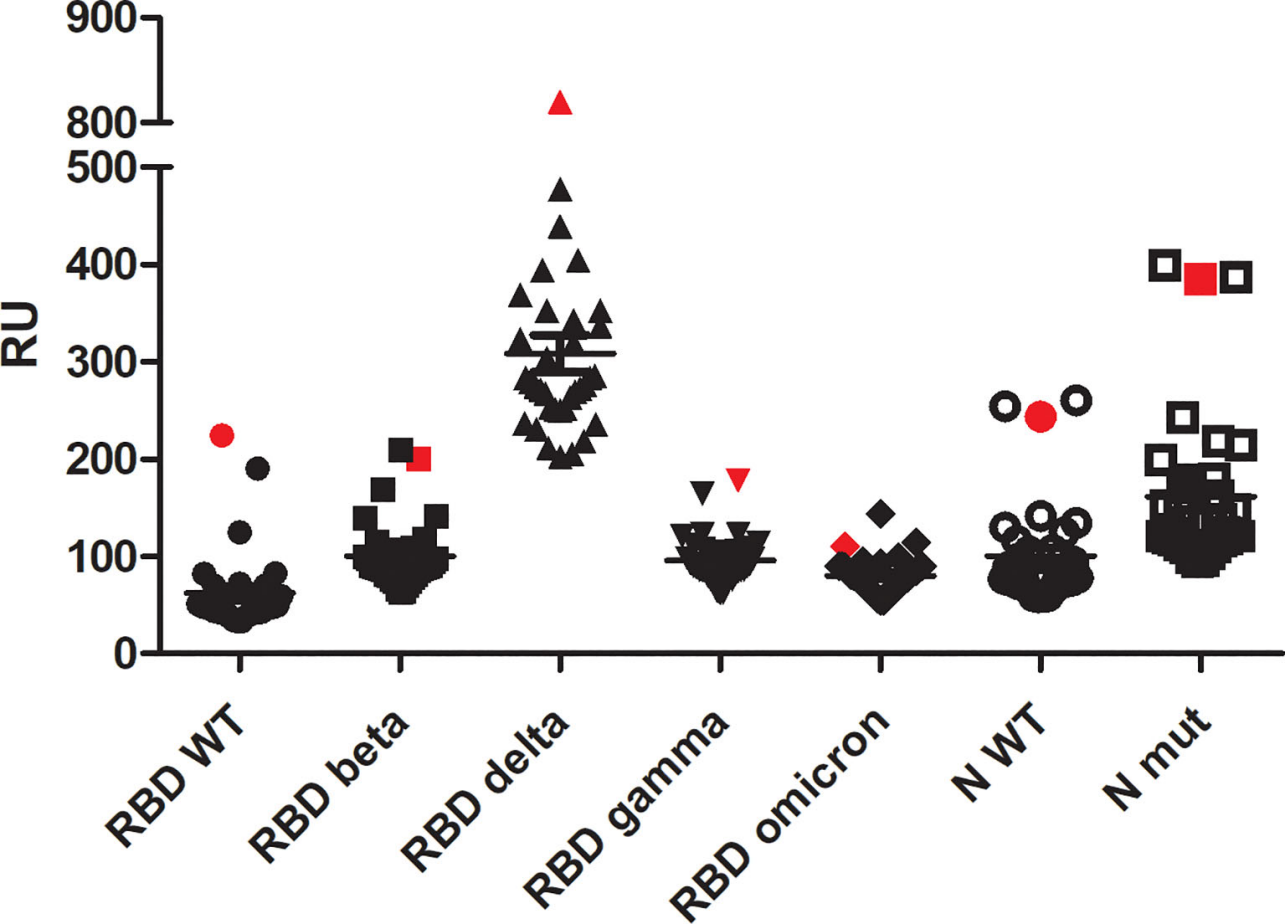
Surface plasmon resonance analysis of ECIG (showed in red symbols) in comparison to 34 sera samples from SARS-CoV-2 infected individuals (not vaccinated). All the samples were diluted at 1:10 v/v in HBS-EP. Binding assays were performed with a BIAcore T200 biosensor instrument, with RBD and N proteins immobilized on a CM5 sensor chip. Binding responses are represented in resonance units (RU). A running buffer was used as a negative control (0 μM).

The binding of ECIG to RBD proteins (wild-type and VOCs) and N proteins (wild-type and mutated) was compared to the sera samples from COVID-19 convalescent individuals collected before the start of vaccination (**Figure 3**).

### Blocking of the ACE2-RBD Interaction by ECIG

**Figure 4:**
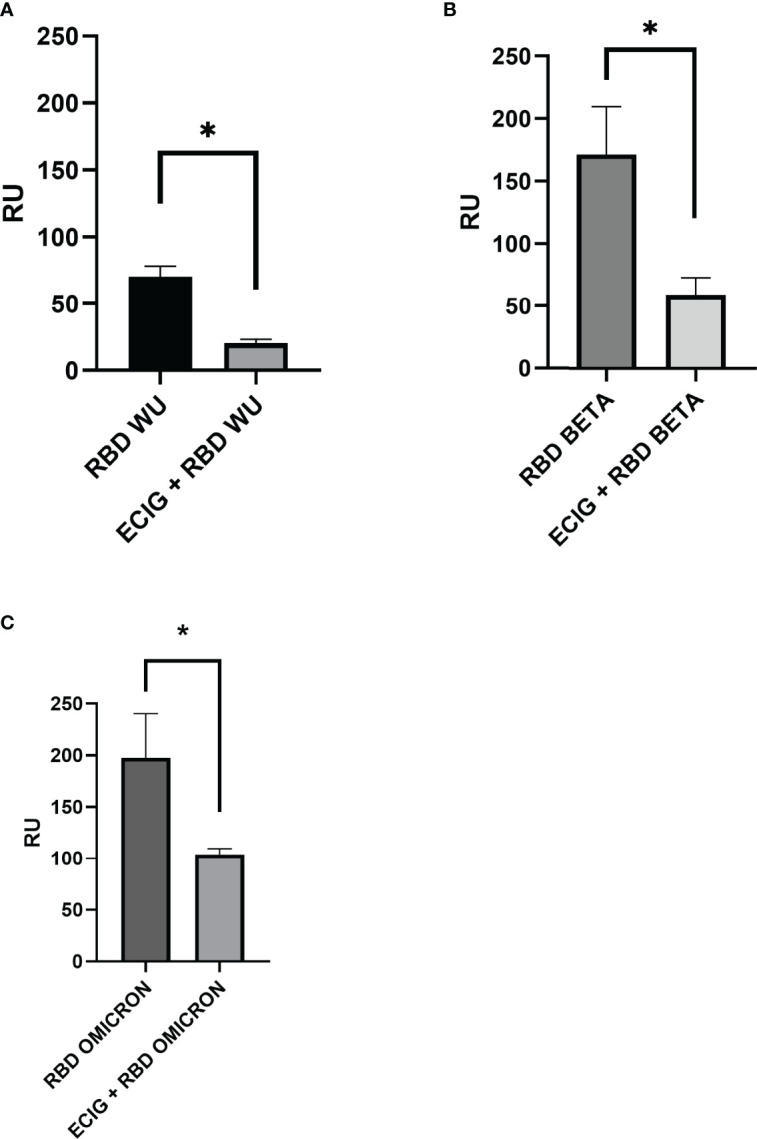
Surface plasmon resonance analysis of **(A)** wild-type RBD, **(B)** RBD Beta, and **(C)** Omicron binding to ACE-2 in the presence and absence of ECIG. At their respective saturation concentration, wild-type RBD, RBD Beta, and RBD Omicron were pre-incubated with ECIG [1:10 v/v] for 1 hour at 37°C. These mixtures were then injected over the surface previously immobilized with ACE-2 to evaluate if ECIG alters SARS-CoV-2 RBD binding to ACE-2. The binding responses are represented in resonance units (RU). Running buffer was used as the negative control (0 μM). Results were evaluated by one-way ANOVA and showed statistical significance (p ≤ 0.05).

Competition assays using human ACE-2 immobilized on the surface of the CM5 biosensor were performed to evaluate whether ECIG inhibits RBD binding to ACE-2. As shown in **Figure 4**, ECIG attenuated wild-type RBD, RBD Beta, and Omicron binding to ACE-2 by 71.2%, 65,8%, and 47,3%, respectively (**Figures 4A–C**). Sensorgrams showing the saturation curves of the binding of RBD WU, Beta, and Omicron to ACE-2 immobilized on the sensor chip and the interference of ECIG on the respective binding can be seen in **Supplementary Figure S2**.

## Discussion

ECIG was prepared from the plasma of horses immunized subcutaneously with four doses of gamma-irradiated SARS-CoV-2 after a study demonstrating its immunogenicity potential while unable to cause disease. Different from chemical inactivation, the radiation maintained the structure of the viral proteins. ECIG is composed of F(ab´)_2_ fragments, thus absent of Fc component, as recommended by Brazilian Pharmacopeia, the case for all equine immunoglobulin preparations produced by Butantan Institute, including anti-virus such anti-rabies serum. Therefore, its neutralization capacity is conferred only by direct virus neutralization, avoiding potential adverse reactions of heterologous Fc-binding. There have been some discussions on the contribution of the Fc moiety towards the neutralization of SARS-CoV-2 and avoiding ADE (antibody-dependent enhancement) in studies conducted with human monoclonal antibodies. Specific antibodies that do not directly neutralize the virus could rely on Fc-effector functions to attain SARS-CoV-2 neutralization. Wrinkler et al. (22) generated human monoclonal antibodies anti-SARS-CoV-2 and reported superior neutralizing activity of intact IgG compared to the loss-of-function mutated IgG. Their results are not similar for all monoclonal antibodies tested; one was not dependent on Fc for protection. In a monoclonal antibody preparation, all the molecules have the same characteristics: binding to the same epitope on the RBD molecule and activating (or inhibiting) the same Fc-effector functions. Another study by Andreano et al. (23) purposely abrogated Fc effector functions by introducing three mutations in the Fc moiety of human monoclonal antibodies. Analyzing a panel of human mAbs, they found a highly neutralizing one that induced prophylactic and therapeutic protection to SARS-CoV-2 in the golden Syrian hamster model. To avoid the potential risk of ADE, only Fc-mutated mAbs were used. The effects of heterologous polyclonal antibodies on the FcR of human cells are not easy to predict and control. The complement system activation should also be avoided. While various studies can be conducted *in vitro* or animal models, only the results of clinical trials could support the conclusions. Hyperimmune equine antibodies have been used for more than a century for different targets. Butantan Institute and other producers digest the equine antibodies with pepsin to cleave them into F(ab)’_2_ fragments (24–26). We consider it safer to rely on the experience of decades.

Based on its extensive experience producing effective hyperimmune sera, the Butantan Institute developed an equine anti-SARS-CoV-2 serum, an antibody product based on the whole virus; inactivated yet structurally preserved viruses were employed for the immunization. Three assays had previously evaluated the neutralization capacity of ECIG. First, the samples were submitted to the current gold standard neutralization method based on a virus neutralization test requiring live pathogen and a biosafety level 3 laboratory, CPE-VNT (cytopathic effect-based virus neutralization test), tested with wild-type and variants of SARS-CoV-2. Three initial preparations of ECIG resulted in neutralization titers of 1:1120-1:2140 for wild-type virus, 1:120-1:200 for gamma variant, and 1:1120-1:2240 for zeta, a VOI that appeared in Brazil, containing E484K/D614G/V1176F mutations (21). Two commercial ELISA kits that correlate with neutralization capacity were conducted in samples of the horse plasma and purified preparation (anti-RBD domain antibody detection kit from ROCHE and cPass Neutralization Antibody Detection kit from GenScript), with consistent results. The final preparation was tested in the golden Syrian hamster model (21).

The entire S protein or S protein fragments, combined with or without other SARS-CoV-2 recombinant proteins, have been used to develop vaccines and therapies. The recombinant RBD protein was used to immunize horses, producing serum used in a clinical trial (27–29). Equine serum produced by immunization with recombinant spike protein was also reported (30). Another group developed equine formulations against S1 or a mixture of recombinant S1, N, and SEM (spike-envelope-membrane mosaic) proteins (31, 32). However, none of these formulations were evaluated by their inhibition of the binding of RBD to ACE-2 receptor, as shown herein.

The S protein contains the RBD that binds to the human ACE-2 receptor and is responsible for virus entry into host cells. Most mutations present in VOCs and VOIs are located in the RBD. Some of these mutations increase the binding affinity to ACE-2 and are associated with increased transmissibility, disease severity, and humoral immunity escape (4). However, other essential viral structural proteins, such as the N protein, are highly immunogenic and play critical roles in viral replication and packaging viral RNA into new virions (33). Besides the S protein mutations, various mutations have been identified in the N protein. It has been reported that after infection, the N protein enters the host cell and facilitates viral replication and virus particle assembly and release (34). The reported substitutions are enriched in the three intrinsically disordered domains: N1a, N2a, and spacer B/N3. There is a particularly high density of substitutions in the SR-rich subdomain of N2a, where R203K and G204R, the most common substitutions, are located (35). Studies have demonstrated that the R203K/G204R mutations improve replication efficiency compared to the original virus. These mutations are dominant during the pandemic, potentially increasing virulence and viral fitness. These multiple mutations and the emergence of new variants have raised concerns about the effectiveness of currently available antibody treatments and the future of the pandemic. ECIG, targeting all proteins of SARS-CoV-2, can be explored as an alternative COVID-19 treatment strategy. The present work assessed the binding of ECIG to SARS-CoV-2 RBD, S1+S2 domains, and N proteins using SPR, a technology widely accepted as the gold standard for characterizing antibody-antigen interactions. The results demonstrated that ECIG recognizes and binds to the RBD present in VOCs. These VOCs display higher virus transmission rates, increasing the risk of reinfection and reducing susceptibility to neutralizing monoclonal antibodies and vaccination. To compare the binding of antibodies present in ECIG in response to immunization of horses with a gamma-irradiated virus to antibodies in the serum of humans convalescent from COVID-19, we performed SPR assays against RBD proteins – wild-type, beta, gamma, delta, and also omicron - as well as N proteins - wild-type and mutated, demonstrating that the range of antibodies present in ECIG is similar as those elicited by the infection with the real virus.

Additionally, ECIG effectively inhibits the binding of wild-type RBD and RBD Beta and Omicron to ACE-2, as demonstrated by our results. The Beta variant is particularly concerning because the RBD contains a set of mutations that may help it evade the neutralization of antibodies raised against the original virus (8, 9). The Omicron variant accumulates many mutations, causing health concerns around the world. In this sense, our results are encouraging because RBD binding to ACE-2 is the central route for SARS-Cov-2 virus infection. Indeed, blocking virus binding to a cell attenuates its spread to other cells, hinders its life cycle, and impairs the illness and course of the disease. Moreover, ECIG’s ability to concomitantly bind to the RBD and N protein increases its neutralizing potential.

From this perspective, the horse serum resulting serum produced by the Butantan Institute for clinical use can effectively treat patients prone to a high risk of complications due to infection with the existing variants. It is also possible that ECIG could be effective against future variants.

## Data Availability Statement

The raw data supporting the conclusions of this article will be made available by the authors, without undue reservation.

## Ethics Statement

The studies involving human participants were reviewed and approved by Committee of Ethics in Research in Human Beings (CEPSH) - Institute of Biomedical Sciences/USP. The patients/participants provided their written informed consent to participate in this study.

## Author Contributions

SAA planned and conducted the experiments, analyzed the data, and wrote the manuscript, JVB-C executed gene design and protein expression, RC contributed with reagents/materials and reviewed the manuscript, FHW contributed to ECIG development, DTC contributed to ECIG development; AMC-T contributed to ECIG development and reviewed the manuscript; AMM planned the experiments, analyzed the data, contributed to the writing of the manuscript and its revision. All authors read and approved the final manuscript.

## Funding

The authors are indebted to FAPESP (2015/15611-0; 2020/07040-1), CNPq (307045/2020-0; 380316/2022-6), and Butantan Foundation for the financial support and fellowships.

## Conflict of Interest

AC-T is co-author of the patent “Processo de produção de um antígeno, correspondente aos vírus SARS-CoV-2 inativado e seus usos”. BR1020200166689, (2020).

The remaining authors declare that the research was conducted in the absence of any commercial or financial relationships that could be construed as a potential conflict of interest.

## Publisher’s Note

All claims expressed in this article are solely those of the authors and do not necessarily represent those of their affiliated organizations, or those of the publisher, the editors and the reviewers. Any product that may be evaluated in this article, or claim that may be made by its manufacturer, is not guaranteed or endorsed by the publisher.
