# Supplementary material for: Equine Anti-SARS-CoV-2 Serum (ECIG) Binds to Mutated RBDs and N Proteins of Variants of Concern and Inhibits the Binding of RBDs to ACE-2 Receptor
Source: Front Immunol. 2022 Jul 11;13:871874. doi: 10.3389/fimmu.2022.871874 (PMC9310548; doi:10.3389/fimmu.2022.871874)
Supplement: Supplementary file 1 [file DataSheet_1.docx]

**Supplementary Material – S1 – nonreducing conditions SDS-PAGE**

Figure S1. Electrophoresis was performed for 90 min at 100 V, and InstantBlue® Coomassie Protein Stain stained the gels. Samples were non-reduced. The characteristics of proteins can be seen in below Table S1.

(A) RBD samples were applied on a 12% gel. Low molecular weight marker from Cytiva, UK.

(B) N Protein applied on a 12% gel. Low molecular weight marker from Cytiva, UK.

(C) Spike proteins were applied on a 7.5% gel. High molecular weight marker from Cytiva, UK.

(D) ACE-2 applied on a 7.5% gel. High molecular weight marker from Cytiva, UK.


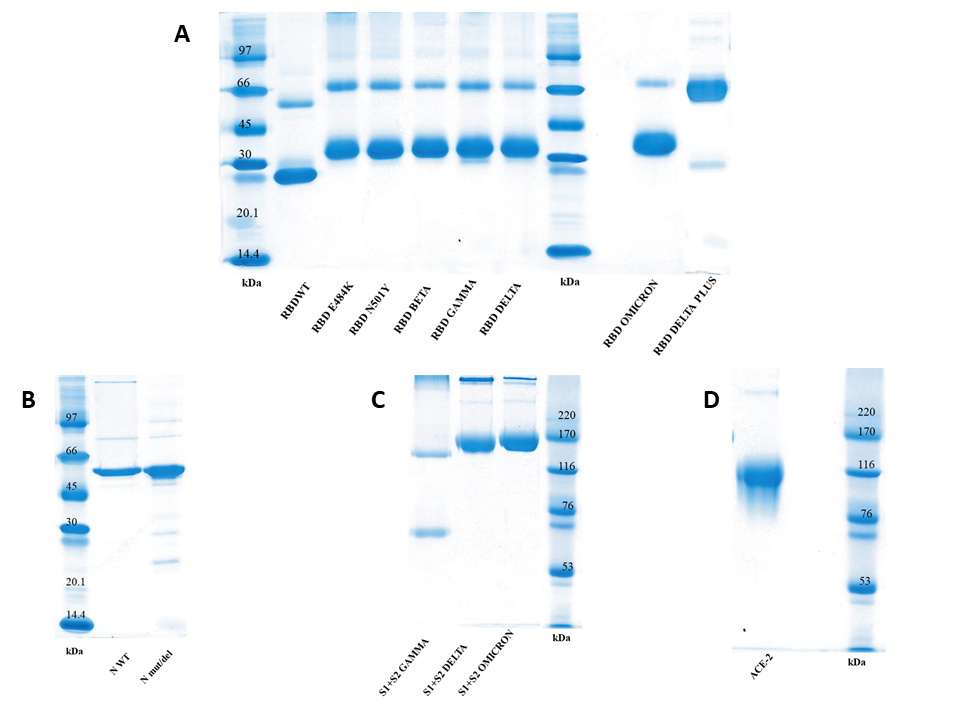


**Supplementary Material S2 - SPR Experiments**

**Part 1 -** Sensorgrams showing the saturation curves of the binding of RBD to ACE-2.

S2a) Sensorgram showing the saturation curves of the binding of RBD WU to ACE-2.


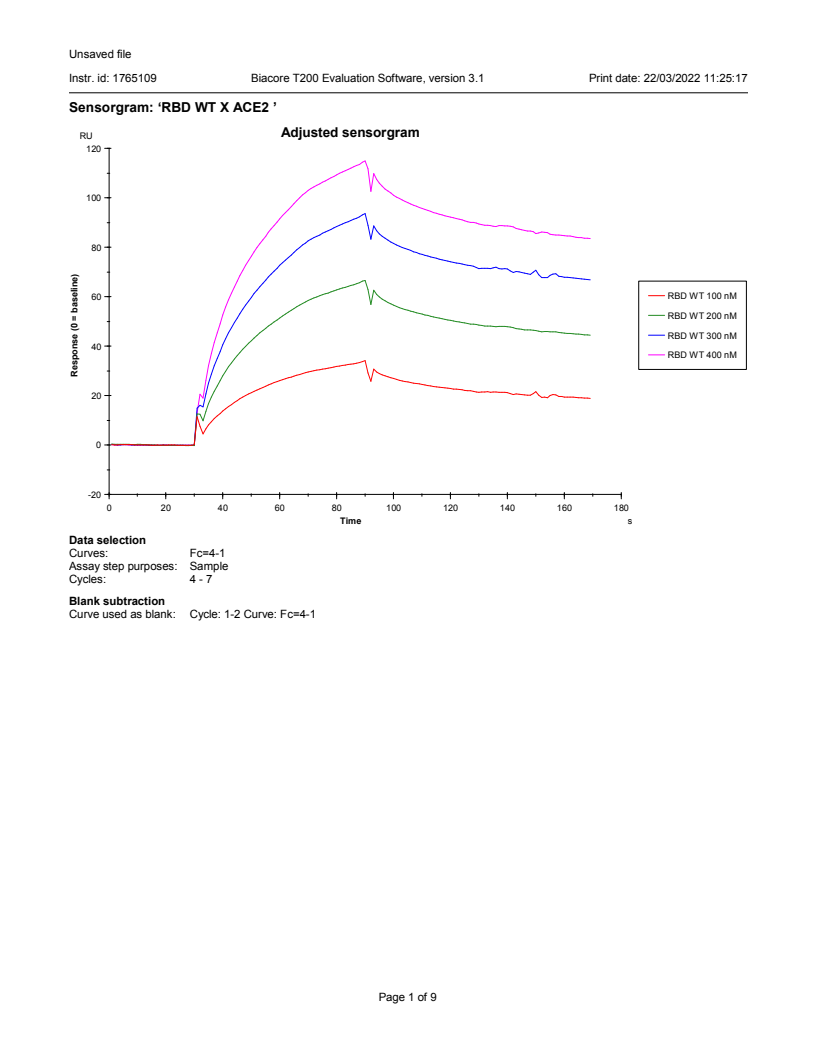
Subtitle: pink (RDB WT 400 nM); blue (RDB WT 300 nM); green (RDB WT 200 nM); red (RDB WT 100 nM)

S2b) Sensorgram showing the saturation curves of the binding of RBD Beta to ACE-2.


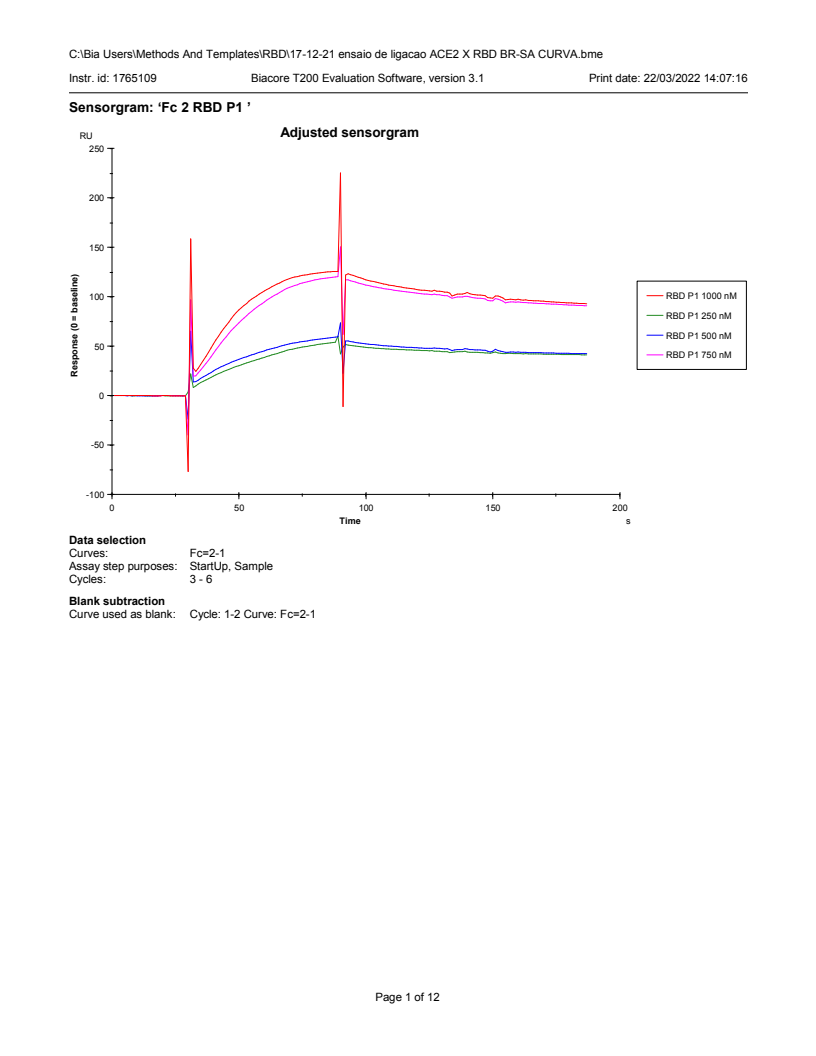
Subtitle: pink (RDB Beta 750 nM); blue (RDB Beta 500 nM); green (RDB Beta 250 nM); orange (RDB Beta 1000 nM).

S2c) Sensorgram showing the saturation curves of the binding of RBD Omicron to ACE-2.

Subtitle: pink (RDB Omicron 750 nM); blue (RDB Omicron 500 nM); green (RDB Omicron 250 nM); orange (RDB Omicron 1000 nM)


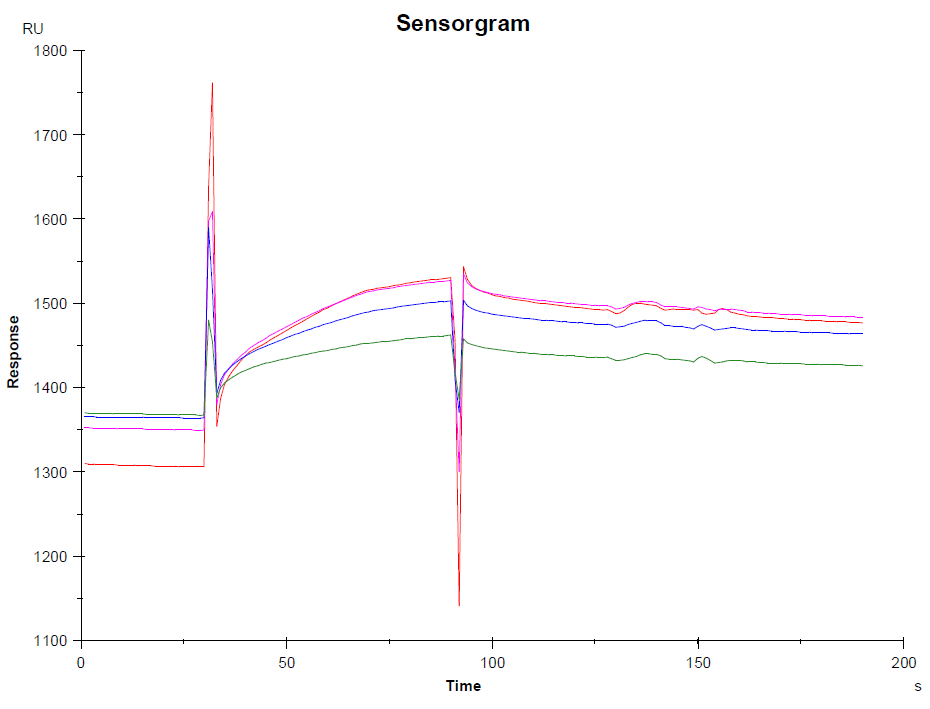


**Part 2** - Sensorgrams showing the interference of ECIG on the RBD

S2a) Sensorgram showing the interference of ECIG on the RBD WT binding


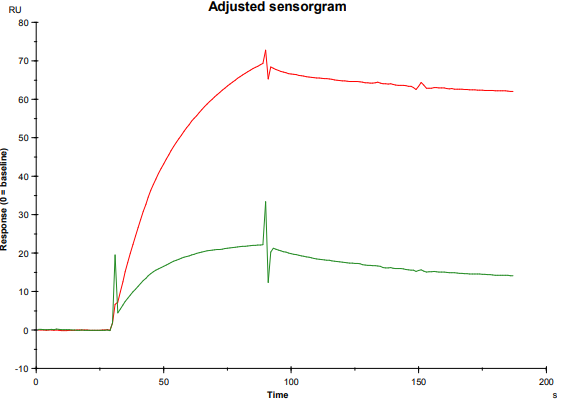


Subtitle: Red (RDB WT 500 nM); green (RDB WT 500 nM + ECIG 1:10 v/v)

S2b) Sensorgram showing the interference of ECIG on the RBD Beta binding


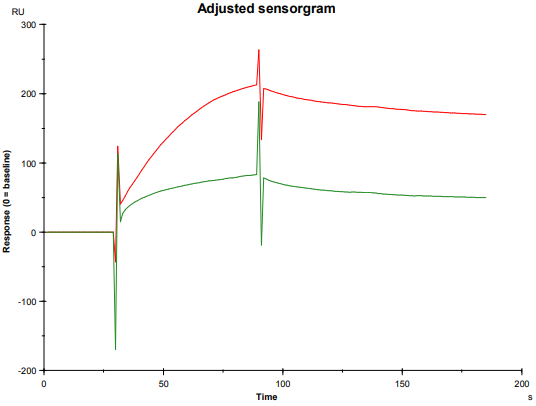


Subtitle: Red (RDB Beta 750 nM); green (RDB Beta 750 nM + ECIG 1:10 v/v)

S2c) Sensorgram showing the interference of ECIG on the RBD Omicron binding


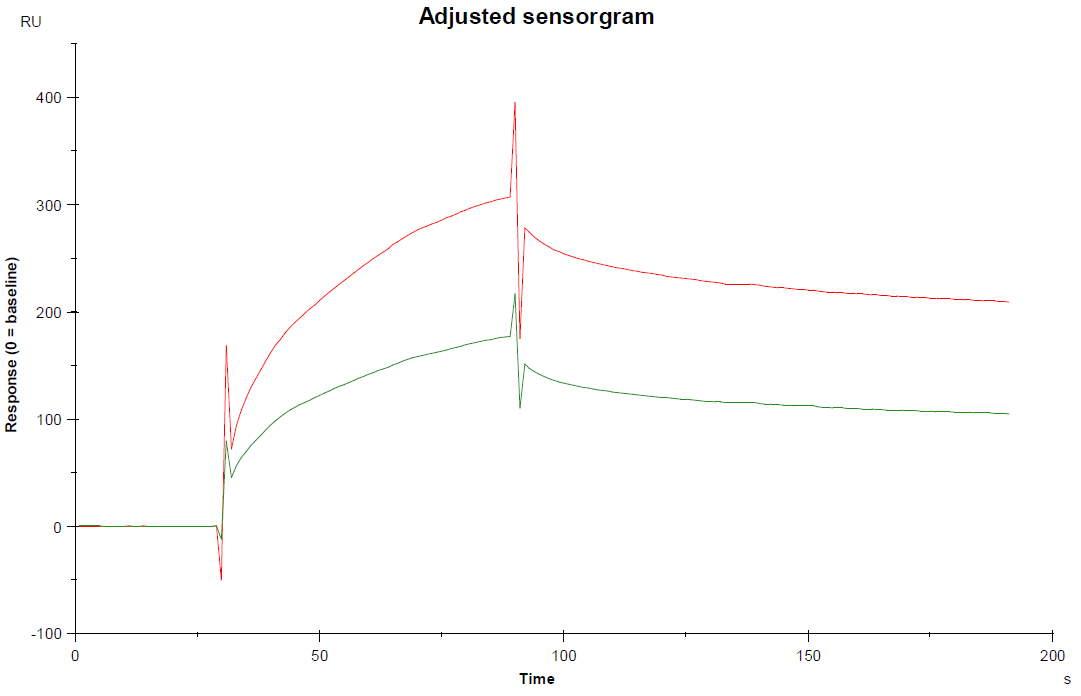


Subtitle: Red (RDB Omicron 750 nM); green (RDB Omicron 750 nM + ECIG 1:10 v/v)
